# Nitrogen-Containing Bisphosphonates Downregulate Cathepsin K and Upregulate Annexin V in Osteoclasts Cultured *In Vitro*

**DOI:** 10.1155/2023/2960941

**Published:** 2023-02-21

**Authors:** Arturo Bautista-Carbajal, Rosina Eugenia Villanueva-Arriaga, Araceli Páez-Arenas, Felipe Massó-Rojas, Nelly Frechero Molina, Salvador García-López

**Affiliations:** ^1^Dental Science Department, Universidad Autónoma Metrolpolitana-Xochimilco, CDMX, Mexico; ^2^Health Science Department, Cell Biology and Immunology Unit, Universidad Autónoma Metropolitana-Xochimilco, CDMX, Mexico; ^3^Translational Medicine Unit, Instituto Nacional de Cardiología “Ignacio Chavez”, CDMX, Mexico

## Abstract

**Introduction:**

Bisphosphonates are widely used in the treatment of osteoporosis; however, they are associated with the serious adverse event of bisphosphonate‐related osteonecrosis of the jaw (BRONJ).

**Aim:**

The aim of this study is to assess the effects of nitrogen-containing bisphosphonates (N-PHs) on the synthesis of IL-1*β*, TNF-*α*, sRANKL, cathepsin K, and annexin V in bone cells cultured *in vitro*.

**Materials and Methods:**

Osteoblasts and bone marrow-derived osteoclasts were cultured *in vitro*, subjected to treatment with alendronate, risedronate, or ibandronate at a concentration of 10^−5^ M for 0 to 96 h and then assayed for IL-1*β*, sRANKL, and TNF-*α* production by ELISA. Cathepsin K and Annexin V-FITC staining in osteoclasts were assessed by flow cytometry.

**Results:**

There was significant downregulation of IL-1*β*, sRANKL, and TNF-*α* in experimental osteoblasts compared to control cells, and there was upregulation of IL-1*β* and downregulation of RANKL and TNF-*α* in experimental osteoclasts. Furthermore, in osteoclasts, cathepsin K expression was downregulated at 48–72 h with alendronate treatment, while risedronate treatment resulted in upregulated annexin V expression at 48 h compared to the control treatment.

**Conclusion:**

Bisphosphonates added to bone cells inhibited osteoclastogenesis, which led to the downregulation of cathepsin K and induction of apoptosis in osteoclasts; these changes limited the capacity of bone remodelling and healing that may contribute to BRONJ induced by surgical dental procedures.

## 1. Introduction

Bisphosphonates (BPs) are widely used in the treatment of osteoporosis and cancer-related bone disease and reduce the incidence of pathological fractures [1]. Oral and intravenous administration of BPs is associated with adverse events of toxicity in the short and long term, including upper gastrointestinal adverse effects, hypocalcaemia, ocular complications, renal failure, and osteonecrosis of the jaws [2]. Moreover, BPs accumulate in human jaws at higher levels than the skeleton [3] and are potent inhibitors of bone resorption [4, 5]. The most efficient BPs have a nitrogen atom in one of the side chains; therefore, they are called nitrogen-containing bisphosphonates (N-BPs). N-BPs include ibandronate, alendronate, and risedronate, among others. These N-BPs decrease bone turnover and prevent bone loss by inhibiting the osteoclast mevalonate pathway [6–11]. Extended treatment with BPs causes severe suppression of bone turnover associated with significant side effects [12, 13]. Bisphosphonate-related osteonecrosis of the jaw (BRONJ), which is characterized by refractory bone exposure, has been recognized as a severe side effect of treatment with BPs. Initially, it was argued that the appearance of BRONJ was due to the intravenous administration of BPs at high doses for the treatment of metastatic bone lesions or multiple myelomas. However, recent studies have indicated that BRONJ also occurs frequently in patients receiving therapeutic oral doses of BPs to treat osteoporosis [14–16].

Studies have shown that BPs inhibit RANKL expression and increase osteoprotegerin (OPG) expression in human osteoblastic cells, inhibiting osteoclast formation [17] and the expression of cathepsin K (CTSK), the main lysosomal protease that degrades type I collagen [9, 18–20]; thus, osteoclasts may express TGF-*β* in the recruitment of osteoblasts [21] and IGF-1 [22] for bone remodelling.

As biofilms are present in the oral environment [23], dental professionals should maintain a healthy oral condition prior to BP therapy; nevertheless, some invasive dental procedures are expected after N-BPs have been prescribed. Although there is no specific rule for the cessation of N-BPs in invasive dental procedures, these procedures must be ceased at least one to three months before and during the healing process [24]. If there was a surgical dental procedure, the administration of amoxicillin-clavulanate (875 mg) and chlorhexidine mouth rinses should be implemented. In cases of allergies to antibiotics, 300 mg of clindamycin should be indicated.

Therefore, the proposed null hypothesis of this study was to evaluate if there is a decrease in the expression of cytokines such as IL-1*β*, TNF-*α*, sRANKL Cathepsin K, and cell apoptosis with the addition of bisphosphonates to *in vitro* cultures of osteoclasts that may affect bone turnover.

## 2. Materials and Methods

### 2.1. Reagents

Alendronate (Fosamax; Merck, Rahway, NY, USA), risedronate (Actonel; Proctor and Gamble, Cincinnati, OH, USA), and ibandronate (Boniva; Roche, Indianapolis, IN, USA) were dissolved in phosphate-buffered saline (PBS) at a concentration of 10^−5^ M.

### 2.2. Murine Osteoblasts

**Figure 1:**
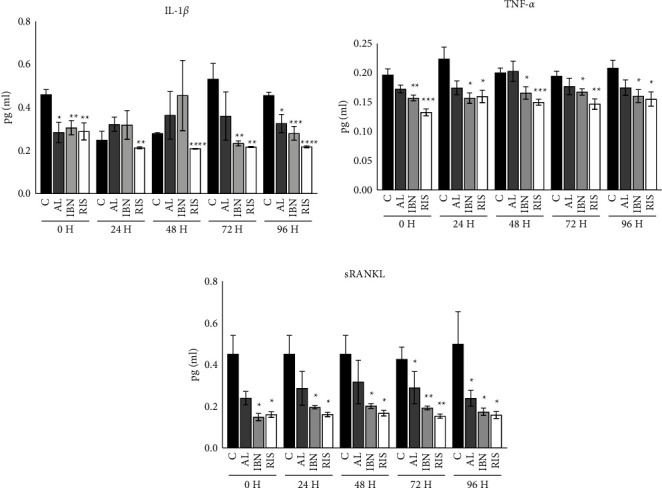
Osteoblast cultures were stimulated with nitrogen-containing bisphosphonates at 10^−5^ M for 0 h, 24 h, 48 h, 72 h, or 96 h, and the culture medium was analysed by ELISA to evaluate IL-1*β*, TNF-,*α* and sRANKL production. Black bars represent the control cell group; AL: alendronate, IBN: ibandronate, and RIS, risedronate. The results are expressed as the mean ± SD of 5 cultures. Significant differences between the experimental and control groups: ^*∗*^*P* < 0.01, ^*∗∗*^*P* < 0.001, ^*∗∗∗*^*P* < 0.001, and ^*∗∗∗∗*^*P* < 0.0001.

Osteoblasts were prepared and characterized by a method previously described by Heath et al. [25] and García-López et al. [26]. The calvaria was carefully dissected from one-week-old neonatal BALB/c mice by removing the adherent soft tissue, washed with a Tyrode solution free of Ca^2+^ and Mg^2+^ and placed in a 30-mm Petri dish. The small bones were cut into smaller pieces with a scalpel blade and sequentially digested with 1 mg/ml trypsin for 20 minutes. The released cells were placed in a falcon tube with 500 mL of medium and centrifuged at 1000 rpm for 5 minutes. The pelleted cells were resuspended in 1 : 1 F12 : Dulbecco's modified Eagle's medium (DMEM) supplemented with 100 units/mL penicillin, 100 mg/mL streptomycin, and 20% foetal calf serum (Gibco, Invitrogen, Carlsbad, CA, USA). Cells were plated in 1.9-cm^2^ 24-well plates (Corning, MA, USA) at a density of 1 × 10^6^ cells/well and cultured at 37°C in a humidified atmosphere of 5% CO_2_/95% air (Figure 1).

### 2.3. Bone Marrow Cells

**Figure 2:**
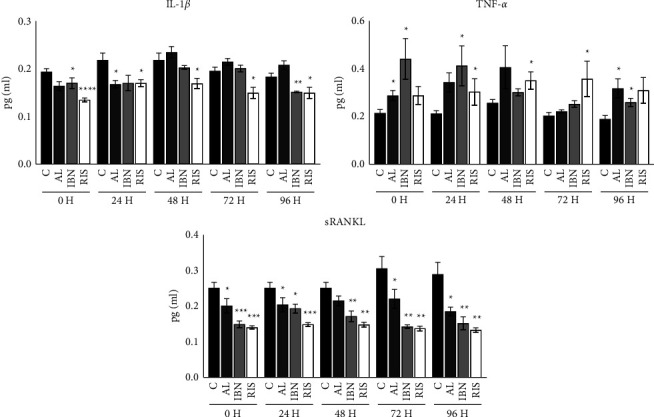
Osteoclastic cultures were stimulated with nitrogen-containing bisphosphonates at 10^−5^ M for 0 h, 24 h, 48 h, 72 h, or 96 h, and the culture medium was analysed by ELISA for IL-1*β*, TNF-*α,* and sRANKL. Black bars represent the control cell group; AL: alendronate, IBN: ibandronate, and RIS: risedronate. The results are expressed as the mean ± SD of 5 cultures. Significant differences between the experimental and control groups: ^*∗*^*P* < 0.01, ^*∗∗*^*P* < 0.001, ^*∗∗∗*^*P* < 0.001, and ^*∗∗∗∗∗*^*P* < 0.0001.

Bone marrow-derived osteoclasts were prepared according to the method previously described by Tumber et al. [27] and García-López et al. [28, 29]. Bone marrow cells were obtained from tibias aseptically removed from one-week-old BALB/c mice; specifically, the tibias were cut into small pieces with a sterile scalpel blade and placed in a 30-mm petri dish with *α*-MEM. The released cells were placed in 12-mm falcon tubes and centrifuged at 1000 rpm for 5 minutes. The pelleted cells were resuspended, plated at 1 × 10^6^ cells/well in 1.9-cm^2^ 24-well plates (Corning, MA, USA) with *α*-MEM supplemented with 100 units/mL penicillin, 100 mg/mL streptomycin, 10^−8^ M 1,25-dihydroxyvitamin *D*_3_ (1,25-(OH)_2_*D*_3_), and 10% foetal calf serum (Gibco, Invitrogen, Carlsbad, CA, USA) and cultured in an incubator (New Air, USA) at 37°C in a humidified atmosphere of 5% CO_2_/95% air (Figure 2).

### 2.4. Tartrate-Resistant Acid Phosphatase Assay

**Figure 3:**
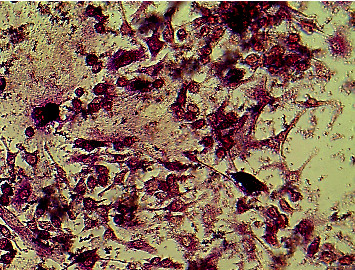
Red TRAP-stained bone marrow-derived cells were imaged with the Plus 2.0 software (Motic Images Plus 2.0, Canada) and an inverted microscope (Motic AE 2000, USA). Three sites were randomly selected and evaluated in each well of a 24-well plate. Positive TRAP staining was quantified as >3 nuclei per cell (220 ± 32.56) using the ImageJ software (ImageJ/Fiji 1.53a, NIH, USA).

Osteoclastic cells were stained to identify tartrate-resistant acid phosphatase (TRAP) following a previous report [29] with a K-*ASSAY* ® TRAP staining kit (Kamiya Biomedical Company, Seattle, WA, USA). Briefly, cells were washed with 100 *μ*L of PBS, fixed with 50 *μ*L of 10% formalin for 5 minutes, and then washed three times with 250 *μ*L of dH_2_O. One vial of the chromogenic substrate (50 *μ*L) was added to each well, and the plates were incubated at 37°C for 20–60 minutes to allow the colour to develop. Finally, the wells were washed with dH_2_O and imaged with an inverted microscope (Motic AE2000, USA) with the New Motic Images Plus 2.0 software (New Motic Images Plus 2.0, BC, Canada). Positive TRAP staining was quantified (>3 nuclei per cell) using the ImageJ software (ImageJ/Fiji 1.53a, NIH, USA) (Figure 3).

### 2.5. Murine Osteoblasts and Osteoclasts Stimulated with BPs

Osteoblasts and osteoclasts were stimulated separately with three different BPs: alendronate, risedronate, and ibandronate (all at 10^−5^ M). Both types of cells were stimulated with a conditioned medium from 0 to 96 h. During the course of the experiments, a 500-*μ*L sample of the supernatant was collected from both the control and experimental groups for 0 h to 96 h. The collected supernatant was stored at −70°C in an ultrafreezer (New Air, USA) for subsequent analysis of IL-1*β*, TNF-*α,* and sRANKL using enzyme-linked immunosorbent assays (ELISAs; R and D Systems, Minneapolis, MN, USA; PeproTech, USA). The absorbance at 450 nm was measured according to the manufacturer's instructions.

### 2.6. Flow Cytometry assay for Cathepsin K

**Figure 4:**
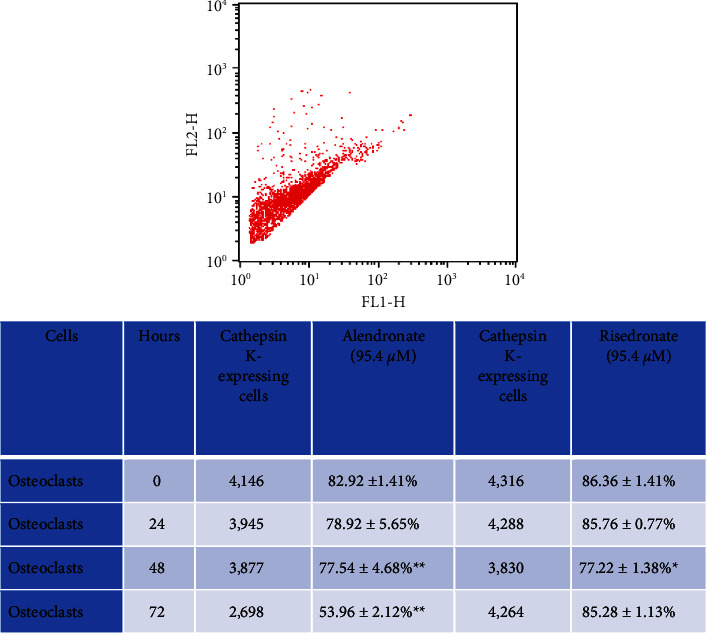
Comparison of cathepsin K expression in cultured osteoclasts stimulated *in vitro* with alendronate (95.4 mM) or risedronate (95.4 mM) with that in control cells. Significant downregulation of cathepsin K at 48 and 72 h with alendronate^*∗∗*^ and at 48 h with risedronate; ^*∗*^*P* < 0.05 and ^*∗∗*^*P* < 0.001. The plot shows flow cytometry staining data.

Osteoclastic culture medium was supplemented with alendronate or risedronate (10^−5^ M) from 0 to 72 h, and a control group was treated with osteoclastic medium without BP stimulation in the presence of 2 brefeldins (BFAs) for four hours. Then, the levels of cathepsin K were evaluated by flow cytometry according to a previous report with modifications [30]. The cellular analysis was performed on a BD FACSCalibur platform (Becton Dickinson, San José, CA), with a total of 5,000 events collected with CellQuest software (Becton Dickinson). Cells were harvested from the culture plate using 0.25% trypsin (Gibco) and centrifuged at 1,500 rpm for 5 minutes. The pelleted cells were washed twice with 1 ml of 1x PBS containing 0.8% bovine serum albumin (BSA) and 0.02% sodium azide. Fixation and permeabilization were performed with an intracellular staining kit (Invitrogen, Carlsbad, CA, USA) following the manufacturer's specifications. Osteoclastic cells were washed in 1 ml of 1x PBS plus BSA. A primary rabbit antihuman cathepsin k polyclonal antibody was incubated with the cells for 30–45 minutes at room temperature; we added a secondary rabbit anti-IgG antibody coupled to phycoerythrin isothiocyanate (PE) for 30–45 minutes at room temperature. A control sample was generated following the same steps for intracellular staining (Figure 4).

### 2.7. Flow Cytometry Assay for Annexin V-FITC Staining

**Figure 5:**
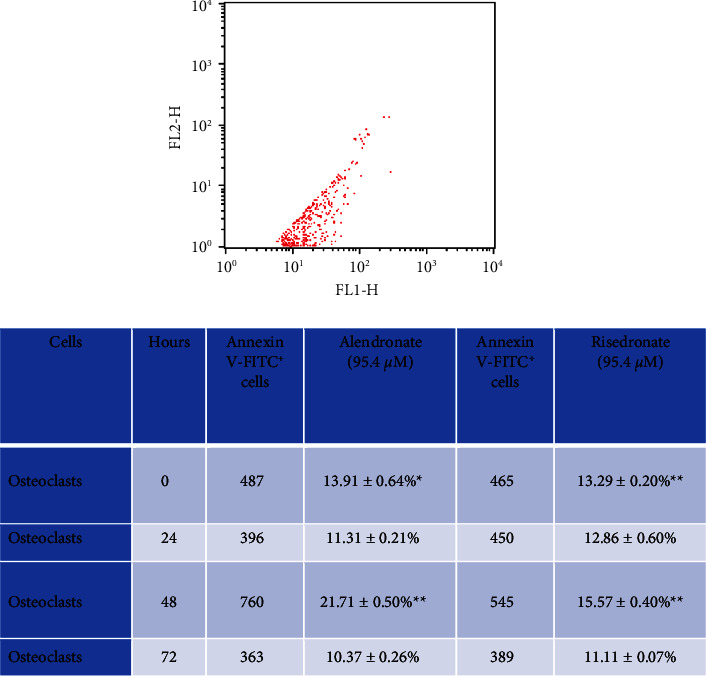
Comparison of Annexin V expression in cultured osteoclasts stimulated *in vitro* with alendronate (95.4 mM) or risedronate (95.4 mM) with that in control cells. Significant upregulation of Annexin V at 0 and 48 h with alendronate^*∗∗*^ and at 0 and 48 h with risedronate; ^*∗*^*P* < 0.05 and ^*∗∗*^*P* < 0.001.

Osteoclast cultures were stimulated with alendronate or risedronate at 10^−5^ M for 0 to 72 h, and a control group without BP stimulation was included. The levels of Annexin V-fluorescein isothiocyanate (FITC) staining were assessed by flow cytometry. Cell analysis was performed on the BD FACSCalibur™ platform (Becton Dickinson, San José, CA), with a total of 5,000 events collected using CellQuest software (Becton Dickinson). Cells were harvested from the culture plate using 0.25% trypsin (Gibco) and centrifuged at 1,500 rpm for 5 minutes. The pelleted cells were washed twice with 1 ml of 1x PBS containing 0.8% BSA and 0.02% sodium azide. Cell staining was performed with an Annexin V-FITC I apoptosis detection kit from BD Pharmingen™ following the manufacturer's specifications. Osteoclastic cells were washed in 1 ml of 1x PBS containing BSA. Two microlitres of Annexin V + FITC and 2 *μ*L of propidium iodide (PI) were added and incubated for 15–30 minutes in the dark at room temperature (24°C). Finally, 400 *μ*L of 1x binding buffer was added for analysis by flow cytometry (Figure 5).

### 2.8. Statistical Analysis

The data are expressed as the mean ± standard deviation (SD). Differences between control and experimental cultures were determined by Student's *t*-test (two tails) using the GraphPad Prism 9 software (GraphPad Software Inc., San Diego, CA, USA.) The level of significance was set at *P* < 0.05.

## 3. Results

(i) Cytokine expression in osteoblasts.
(ii) Cytokine expression in osteoclasts.
  (iii) TRAP expression in bone marrow cells.
(iv) Comparison of cathepsin K expression in osteoclasts cultured *in vitro* with BP stimulation evaluated by the flow cytometry.

(v) Comparison of annexin V (FITC) expression in osteoclasts cultured *in vitro* with BP stimulation by the flow cytometry.

## 4. Discussion

This study evaluated the effects of N-BPs on primary bone cell cultures at a dose of 10^−5^ M, which is within the therapeutic range according to Chen et al. [26], for 0–72 hours as described in previous studies [26, 28, 29].

During the course of the experiments with osteoblasts in culture, constitutive production of IL-1*β*, TNF-a, and RANKL was observed in both types of cells. The production of IL-1*β*, TNF-a, and IL-6 by osteoblasts synergistically enhances RANKL [28, 29] and may also contribute to the stimulation, differentiation, and formation of bone. Therefore, peripheral blood monocytes differentiate into osteoclasts by signalling through their receptor RANKr, and large amounts of RANKL have to be expressed by osteoblasts to differentiate mature osteoclasts, consolidate the osteoclastogenic process to initiate bone resorption, maintain the homeostatic activity coordinated by osteoblasts, and induce the expression of OPG, the natural decoy of RANKL that inhibits osteoclastogenesis, an effect mediated by the expression of IL-4 and IL-13 [26]. IL-17 is also expressed by bone cells to stimulate CatK to degrade bone matrix, which stimulates periostin and thereby moderates Wnt-*β*-catenin signalling to induce cortical bone formation [31].

This study showed downregulation followed by upregulation and then downregulation of IL-1*β* expression with alendronate or ibandronate treatment, while there was only downregulation with risedronate treatment in osteoblast cultures. The N-BPs alendronate and ibandronate produced alterations in IL-1*β* at a therapeutic dose, and it has been shown that low concentrations of N-BPs (10^−3^ M) may increase cell proliferation and reduce the proliferation of osteoblasts, while high doses (10^6−10^ M) may induce apoptosis [32–34]. In osteoclasts, there was upregulation of IL-1*β* with treatment with any of the three N-PHs at a therapeutic dose (10^−5^ M), which may induce proliferation of these cells and activate RANKL, as seen at low concentrations [35, 36]. Nevertheless, there was also downregulation of TNF-a in the experimental group of osteoblast cultures with each of the three N-PHs, which is consistent with other studies [37, 38]. The downregulation and upregulation observed in the experimental groups of osteoclasts treated with alendronate or ibandronate and the downregulation observed with risedronate treatment could be due to the accumulation or consumption of N-BPs, which may enhance TNF-a production or inhibit osteoclast formation [39].

In relation to the expression of RANKL, both osteoclasts and osteoblasts showed downregulation of RANKL. Thus, taken together with the downregulation of IL-1*β* and TNF-*α*, the study results show that N-PHs produce an inhibitory effect on the osteoclastogenic process, which may produce the side effects of inhibiting bone resorption as a result of osteoclast apoptosis. Therefore, there is no stimulation of bone remodelling or only low remodelling, which may result in fractures in the bone to contribute to BRONJ [9]. Moreover, another study showed that at high concentration of 5 × 10^−5^ M, N-PHs may enhance OPG gene expression and strongly increase that of RANKL [40].

Furthermore, cathepsin K and annexin V were evaluated in only osteoclast cultures treated with alendronate or risedronate; nonetheless, these results, similar to those of another study [41], demonstrated that alendronate and risedronate at a dose of 10^−5^ M inhibited osteoclast proliferation and induced apoptosis *in vitro* [34, 42].

Cathepsin K is expressed at high levels in osteoclasts and functions as a protease, being the primary enzyme responsible for the degradation of type I collagen, which composes the organic matrix; cathepsin K is able to completely dissolve human cortical bone [43]. Nevertheless, cathepsin K was downregulated in osteoclast cultures by the N-PHs studied, which suggested a lack of matrix degradation, as cathepsin K production by osteoclasts should induce bone remodelling stimulated by periostin degradation [31, 44, 45].

The evaluation of Annexin V-FITC staining in the osteoclast culture group showed significant upregulation in the experimental group compared to the control group, as it is known that BPs induce osteoclast apoptosis via the inhibition of farnesyl pyrophosphate synthase through the mevalonate pathway, which produces prenylation in osteoclasts that contributes to the inhibition of bone resorption by disrupting osteoclast function [8]. However, it has been shown that apoptotic bodies derived from osteoclasts possess osteogenic potency mediated by activating RANKL reverse signalling in preosteoblasts [46] to stimulate osteoblasts and other cells around the bone; therefore, it has been suggested that the administration of calcitonin in conjunction with N-PHs may prolong osteoclast survival by inhibiting apoptosis [47].

The effects of apoptosis in endothelial cells induced by BPs and the impaired viability of fibroblasts and oral keratinocytes may induce vascular damage and produce a lack of circulation in the jawbones, which affects bone healing. Likewise, the presence of *Actinomyces* species in significant numbers led Naik and Russo [48] to suggest a critical role for these organisms, among other factors, in the development of BRONJ.

Within the limitations of our *in vitro* study, these findings confirm our hypothesis, and we concluded from a clinical standpoint that murine osteoblasts and bone marrow-derived osteoclasts stimulated with a therapeutic dose of N-PHs (10^−5^ M) showed inhibition of IL-1*β*, TNF-*α*, and RANKL, although IL-1*β* and TNF-*α* contribute to the expression of RANKL, which stimulates osteoclast activity. This was inhibited by the N-PHs and there was also downregulation of cathepsin K. Therefore, there is no stimulation of periostin to activate cortical bone formation and the stimulation of apoptosis in osteoclasts, which does not allow the development of mature osteoclasts to induce bone resorption. These changes limited the capacity of bone remodelling and healing that may contribute to BRONJ induced by surgical dental procedures, among other factors.

## Data Availability

The data that support the findings of this study are available from the corresponding author upon reasonable request.
